# Comparison of anal function and quality of life after conformal sphincter preservation operation and intersphincteric resection of very low rectal cancer: a multicenter, retrospective, case–control analysis

**DOI:** 10.1007/s10151-023-02819-w

**Published:** 2023-05-29

**Authors:** G. Sun, Y. Zang, H. Ding, Y. Chen, D. Groothof, H. Gong, Z. Lou, R. Meng, Z. Chen, E. Furnee, J. Xiang, W. Zhang

**Affiliations:** 1 Department of Colorectal Surgery, Changhai Hospital, Naval Medical University, 168 Changhai Road, Yangpu District, Shanghai, 200433 China; 2 Department of General Surgery, Huashan Hospital, Fudan University, Shanghai, 200040 China; 3 Department of Surgery, University Medical Center Groningen, Groningen, The Netherlands; 4 Department of Epidemiology and Public Health, University College London, London, UK; 5 Department of Internal Medicine, University Medical Center Groningen, Groningen, The Netherlands

**Keywords:** Conformal sphincter preservation operation, Intersphincteric resection, Anal function, Quality of life, Very low rectal cancer, Outcome

## Abstract

**Purpose:**

Conformal sphincter preservation operation (CSPO) is a sphincter preservation operation for very low rectal cancers. Compared to intersphincteric resection (ISR), CSPO retains more dentate line and distal rectal wall, and also avoids damaging the nerves in the intersphincteric space. This study aimed to compare the postoperative anal function and quality of life between the CSPO and ISR.

**Method:**

Patients with low rectal cancer undergoing CSPO (*n* = 117) and ISR (*n* = 66) were included from Changhai and Huashan Hospital, respectively, between 2011 and 2020. A visual analog scale (range 0–10) was utilized to evaluate satisfaction with anal function and quality of life. The anal function was evaluated with Wexner scores and low anterior resection syndrome (LARS) score. Quality of life was evaluated with the EORTC QLQ-C30 and QLQ-CR38.

**Results:**

The CSPO group had more male patients (65.8% vs. 50%, *p* = 0.042), more preoperative chemoradiotherapy (33.3% vs. 10.6%, *p* < 0.001), lower tumor position (3.45 ± 1.13 vs. 4.24 ± 0.86 cm, *p* < 0.001), and more postoperative chemotherapy (65% vs. 13.6%, *p* < 0.001) compared to the ISR group. In addition, CSPO patients had shorter postoperative stay (6.63 ± 2.53 vs. 7.85 ± 4.73 days, *p* = 0.003) and comparable stoma reversal rates within 1 year after surgery (92.16% vs. 96.97%, *p* = 0.318). Multivariable analysis showed that CSPO significantly contributed to higher satisfaction with anal function (beta = 1.752, 95% CI 0.776–2.728) and with quality of life (beta = 1.219, 95% CI 0.374–2.064), but not to Wexner, LARS score, or EORTC QLQ-C30 and QLQ-CR38.

**Conclusion:**

CSPO improved the satisfaction with anal function and quality of life but utilized more preoperative chemoradiotherapy. CSPO may be an alternative choice for patients with very low rectal cancers in better physical health and with higher requirements for anal function and quality of life.

**Supplementary Information:**

The online version contains supplementary material available at 10.1007/s10151-023-02819-w.

## Introduction

Intersphincteric resection (ISR) is a sphincter preservation operation for very low rectal cancers. However, according to the literature and our experience with ISR, patients often have poor postoperative anal function, leading to decreased quality of life. This might be due to partial or total resection of the internal anal sphincter and dentate line, but also due to damaging the autonomic nerves during extensive dissection in the sphincteric space [1–3]. To improve postoperative anal function for patients with very low rectal cancer, we designed the conformal sphincter preservation operation (CSPO). CSPO retains more dentate line and distal rectal wall and also protects the autonomic nerve by avoiding dissection in the sphincteric space. In addition, the anastomosis is fashioned on the part with more rectal wall preserved, thus the anastomosis can be 2–3 cm above the dentate line to get more satisfactory anal function after resection. The preliminary experience of this operation has been published previously [4–6]. The oncological outcome and perioperative safety, including postoperative complications, were already found to be comparable between CSPO and ISR according to our previous study [7]. However, anal function and quality of life after CSPO are still unknown in comparison to ISR, even though functional outcome and quality of life are playing a more and more important role in the evaluation of surgical procedures.

This study aimed to compare the anal function and quality of life between the CSPO and ISR.

## Patients and methods

### Patient selection

The clinical data of patients who underwent CSPO and ISR for very low rectal cancer was collected in Changhai Hospital Affiliated to Naval Medical University (*n* = 117) and Huashan Hospital Affiliated to Fudan University (*n* = 66), respectively, from August 2011 to April 2020. This study was approved by the Ethics Committee of the First Affiliated Hospital of Naval Medical University (committee’s reference number CHEC2022-021) and followed the precepts established by the Helsinki Declaration. Each patient signed the informed consent. The work has been reported in line with the STROCSS criteria [8]. This research was retrospectively registered in the Chinese Clinical Trial Register (ChiCTR2300070971).

The inclusion and exclusion criteria for patients undergoing CSPO were described previously by Sun et al. [4]. The inclusion criteria are briefly described as follows: (1) diagnosis of rectal adenocarcinoma by digital rectal examination, colonoscopy, and biopsy; (2) the tumor does not infiltrate the intersphincteric space; (3) good anal function before surgery evaluated by Wexner incontinence score in combination with the digital rectal examination during consultation; (4) distance between the lower tumor edge and the dentate line is within 2 cm or the distance of the lower tumor edge from the anal verge is less than 4–5 cm; (5) the diameter of the tumor is less than 3 cm with no more than 1/3 circumference of the intestinal lumen; (6) the American Society of Anesthesiologists Score (ASA) is ≤ 3.

The following patients were excluded: (1) Distant metastasis (including lymph node metastasis outside of the mesorectum); (2) patients not able to tolerate the operation (ASA > 3).

### Surgical methods

All CSPO procedures were conducted at Changhai Hospital, while all ISR procedures took place at Huashan Hospital. The surgeons performing ISR or CSPO were experienced colorectal surgeons with similar levels of expertise. Both hospitals are tertiary institutions situated in the same city, Shanghai. All clinical treatments adhered to the same guidelines. We give patients preoperative chemoradiotherapy in case of preoperative stage T3–T4 or N+, or mesorectal fascia (MRF) involvement in MRI assessments after communication with patients.

### CSPO procedures

**Fig. 1:**
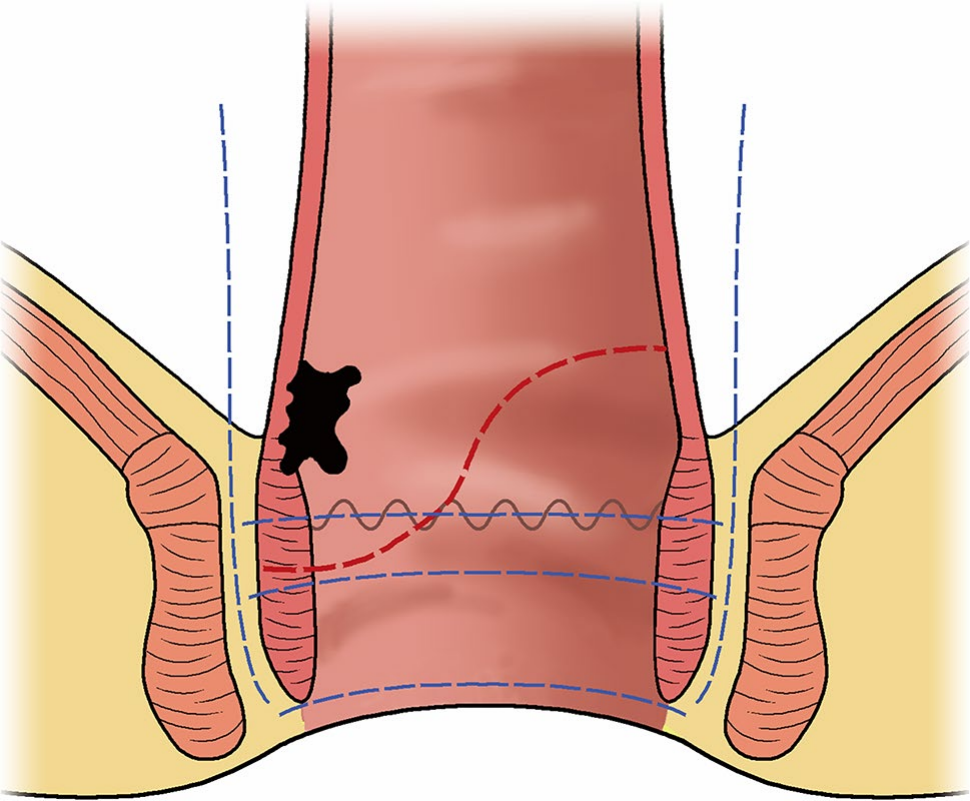
Surgical procedures for CSPO (red line); total, subtotal, and partial ISR (blue lines). Adapted with permission from Sun et al. [4]

The key steps of CSPO were as follows [4, 5]: the sigmoid colon was mobilized in the standard manner to decrease the tension during the later anastomosis. The origin of the inferior mesenteric artery was ligated. The rectum was dissected according to the TME (total mesorectal excision) principle with autonomic nerve preservation. The rectum was dissected up to the hiatal ligament which is the sequence of the anococcygeal raphe body [9]. After the hiatal ligament was cut off, further dissection into the intersphincteric space was not performed to prevent damage to the nerve structure, which is different from ISR. The intestine was transected at the rectosigmoid junction with the proximal resection margin length > 15 cm. The anus was dilated to 3–4 fingers wide, through which the rectum was pulled out. In case of difficult eversion from the rectum due to a fatty mesorectum or a narrow pelvic cavity, transanal resection like TaTME (transanal total mesorectal excision) was utilized without eversion of the rectum. A conformal resection line was designed according to the position and shape of the tumor: the rectal wall, dentate line, and internal anal sphincter were retained as much as possible on the opposite side of the tumor (Fig. 1). The distal resection margin length was at least 1 cm under direct vision. For patients who received preoperative chemoradiotherapy, we maintained a distal resection margin length longer than 1 cm, as observed with the naked eye during the operation [10]. Intraoperative frozen pathological examination was utilized to ensure a safe distal resection margin. The rectal stump was closed manually with interrupted sutures. A 25-mm circular stapler was inserted through the anus, and the anastomosis was made on the side with more rectal wall, dentate line and internal sphincter retained. In case the left rectal stump was too short to insert a circular stapler, manual anastomosis was used. For the manual anastomosis, absorbable threads (size 3–0) were utilized to make four sutures at the top, bottom, left, and right sides. After fixation of the four sutures, two 3–0 sutures with barbs were used in succession, with each suturing half a circle. A prophylactic ileostomy was created routinely.

### ISR procedures

In the ISR procedures, first, the sigmoid colon was mobilized in the standard manner to decrease the tension for later anastomosis. After TME was completed and the anococcygeal raphe was cut off transabdominally, the intersphincteric space was mobilized through the pelvic approach, exposing the puborectalis muscle, cutting the hiatal ligament, and entering the intersphincteric space through the lateral and posterior approaches and dissecting for about 2–4 cm caudally. Some joint longitudinal muscles in the sphincteric space were cut off. The anus was dilated and exposed with a Lonestar retractor.

A purse string was sutured two times just distal to the lower edge of the tumor to close the bowel. The distal rectum was irrigated. A circular incision was made at about 1–1.5 cm distal to the purse-string suture, to cut off the internal sphincter and joint longitudinal muscle, and meet the other dissection from the pelvic approach. The rectosigmoid was transected at the rectosigmoid junction. The specimen was removed through the anus. The proximal sigmoid colon and the anal canal were anastomosed by interrupted sutures (approximately 24–32 stitches). All patients had a prophylactic ileostomy.

### Data collection

The following patient characteristics were collected: age, sex, body mass index (BMI), tumor diameter, tumor position (the distance between the lower edge of the tumor and the anal verge), clinical T stage, clinical N stage, preoperative chemoradiotherapy, postoperative chemotherapy, pathological T stage, pathological N stage, and tumor differentiation.

The following surgery-related information was collected: blood loss, surgical approach, operation duration, postoperative hospital stay, distal resection margin length, and the number of retrieved lymph nodes.

### Anal function evaluation

The anal function after ileostomy reversal was evaluated with the Wexner incontinence score (range 0–20, where 0 indicated perfect continence, and 20 indicated the most severe form of fecal incontinence), low anterior resection syndrome score (LARS score, range 0–42, where 0–20 indicated no LARS, 21–29 minor LARS, 30–42 major LARS), and visual analog scale of anal function satisfaction (VAS, range 0–10, where 0 indicated the lowest satisfaction, 10 indicated the highest satisfaction) [11–13].

### Quality of life evaluation

VAS was also utilized to evaluate satisfaction with the quality of life [14, 15] (range 0–10, 0 indicating the lowest satisfaction and 10 the highest satisfaction). Detailed domains of quality of life were evaluated with the EORTC QLQ-C30 questionnaire [16] in combination with the EORTC QLQ-CR38 [17] questionnaire.

The EORTC QLQ-C30 questionnaire includes the following domains: global health (QL2), physical function (PF2), role function (RF2), emotional function (EF), cognitive function (CF), social function (SF), fatigue (FA), nausea and vomiting (NV), dyspnea (DY), pain (PA), insomnia (SL), appetite loss (AP), constipation (CO), diarrhea (DI), and financial difficulties (FI).

The EORTC QLQ-CR38 questionnaire includes the following domains: body image (BI), sexual function (SX), sexual enjoyment (SE), future perspective (FU), micturition problem (MI), chemotherapy side effect (CT), symptoms of gastroenterology (GI), male sexual problem (MSX), female sexual problem (FSX), stoma-related problem (STO), and weight loss (WL).

All the above domains in C30 and CR38 have a range between 0 and 10. A score of 0 in a particular functional domain indicates the worst function, and a score of 0 in symptom domains indicates the least severe symptoms. A score of 100 in functional domains indicates the best function, and a score of 100 in symptom domains indicates the most severe symptoms.

### Statistical analysis

We used R software (version 1.4.1106, 2021) to analyze data and generate figures. Continuous variables were presented as mean ± standard deviation or median and interquartile ranges depending on whether the data were normally distributed or not. Categorical variables were analyzed with the chi-square test, and continuous data were compared with a Student’s *t* test or Mann–Whitney *U* test depending on the distribution of the data.

The Kaplan–Meier curve was used to describe the time to ileostomy reversal. Cox regression analysis was used to analyze risk factors for ileostomy reversal time between the operation and reversal. Multivariable linear regression was used to analyze factors affecting satisfaction with anal function and quality of life, and their specific domains, i.e., Wexner score, LARS score, EORTC-C30, and EORTC-CR38. For the binary categorical outcome, we used the logistic regression analysis.

The following factors which may affect ileostomy reversal, postoperative anal function, or quality of life were first included in the univariable linear regression analysis: type of operation, age, gender, BMI, tumor position, tumor diameter, tumor differentiation, pathological stage, preoperative chemoradiotherapy, postoperative chemotherapy. In the analysis of anal function and quality of life, whether ileostomy reversal was performed more than 12 months ago or not was included as a variable in the univariable regression analysis as well. Variables with a *p* value smaller than 0.15 in univariable analysis were selected to be included in multivariable regression analysis using the backward elimination method. A *p* value less than 0.05 (two-sided) was considered statistically significant in multivariable analysis.

## Results

### Enrollment of patients

**Fig. 2:**
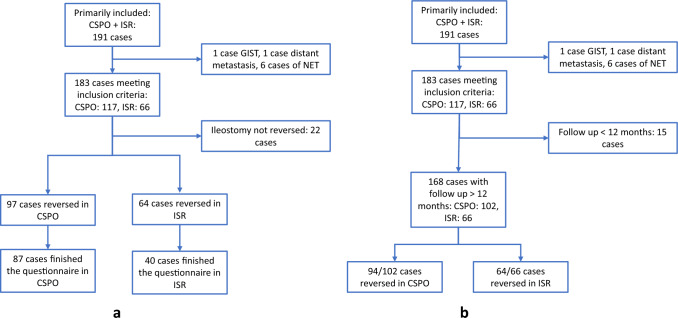
Patient flowchart

The enrollment of patients is shown in Fig. 2a. There were overall 123 cases of CSPO during the study period, of which 6 cases were excluded (including 4 cases of neuroendocrine tumors, 1 case of preoperative distant metastasis, 1 case of stromal tumor), and finally, 117 cases of CSPO were included for the current study. There were overall 68 cases of ISR, and 2 cases of neuroendocrine tumors were excluded. Finally, 66 patients with ISR were included.

### Clinicopathological data

The basic characteristics, i.e., age, BMI, tumor diameters, cT stage, cN stage, pT stage, pN stage, tumor differentiation, were not significantly different between the CSPO and ISR groups (Table 1). The CSPO group had a significantly higher percentage of male patients (*p* = 0.042), a lower tumor position (*p* < 0.001), more preoperative chemoradiotherapy (*p* = 0.001), and a higher percentage of postoperative chemotherapy (*p* < 0.001) in comparison to ISR (Table 1).

**Table 1:** Clinical and pathological data

Variable	CSPO (*n* = 117)	ISR (*n* = 66)	p*
Age (years)^a^	57.52 ± 10.88	57.08 ± 10.61	0.789
Male (%)	77 (65.8%)	33 (50%)	0.042^#^
BMI (kg/m^2^)^a^	22.94 ± 3.08	23.74 ± 2.98	0.089
Tumor position (cm)^a,b^	3.45 ± 1.13	4.24 ± 0.86	< 0.001^#^
Preoperative chemoradiotherapy			0.001^#^
Yes	39 (33.3%)	7 (10.6%)	
No	78 (66.7%)	59 (89.4%)	
Blood loss (ml)^a^	141.75 ± 100.70	91.21 ± 85.46	0.001^#^
Laparoscopic approach (%)	44 (37.6%)	55 (83.3%)	< 0.001^#^
Operation duration (min)^a^	164.82 ± 53.77	267.80 ± 101.19	< 0.001^#^
Distal resection margin length (cm)^a^	0.62 ± 0.41	1.93 ± 0.58	< 0.001^#^
Number of retrieved lymph nodes^a^	12.38 ± 4.65	12.21 ± 4.80	0.812
Tumor diameter (cm)^a^	2.90 ± 1.31	2.93 ± 1.34	0.909
cT			0.324
T1	17 (14.4%)	7 (10.6%)	
T2	57 (48.7%)	29 (43.9%)	
T3	37 (31.6%)	11 (16.7%)	
T4	1 (0.9%)	0	
Unknown	5 (4.3%)	19 (28.8%)	
cN			0.410
N0	60 (51.3%)	28 (42.4%)	
N1–2	52 (44.5%)	20 (30.3%)	
Unknown	5 (4.3%)	18 (27.3%)	
pT			0.743
T0	7 (6%)	2 (3%)	
T1	22 (18.8%)	9 (13.6%)	
T2	56 (47.9%)	40 (60.6%)	
T3	32 (27.4%)	15 (22.7%)	
pN			0.117
N0	95 (81.2%)	48 (72.7%)	
N1–2	22 (18.8%)	18 (27.3%)	
Tumor differentiation			0.067
High	9 (7.7%)	1 (1.5%)	
Moderate	98 (83.8%)	56 (84.8%)	
Low	10 (8.5%)	9 (13.6%)	
Postoperative hospital stays (days)^a^	6.63 ± 2.53	7.85 ± 4.73	0.003^#^
Postoperative chemotherapy			< 0.001^#^
Yes	76 (65%)	9 (13.6%)	
No	41 (35%)	57 (86.4%)	

CSPO performed in Changhai Hospital, ISR performed in Huashan Hospital

*Difference between CSPO and ISR

^#^*p* value < 0.05

^a^Mean ± standard deviation

^b^Lower edge of the tumor to the anal verge

### Surgery-related information

No patients received pouch reconstruction in either CSPO or ISR group. The CSPO group had significantly more patients operated on with an open approach (*p* < 0.001), including one case converted from laparoscopic to open surgery. The CSPO group also had more blood loss (*p* = 0.001), shorter operation duration (*p* < 0.001), shorter distal resection margin length (*p* < 0.001), and shorter postoperative hospital stays (*p* = 0.003) than the ISR group. The number of retrieved lymph nodes during the operation was not significantly different between both groups. All patients in both groups achieved R0 resection. All patients had negative circumferential resection margin (CRM) and complete total mesorectal excision (TME).

### Stoma reversal

**Fig. 3:**
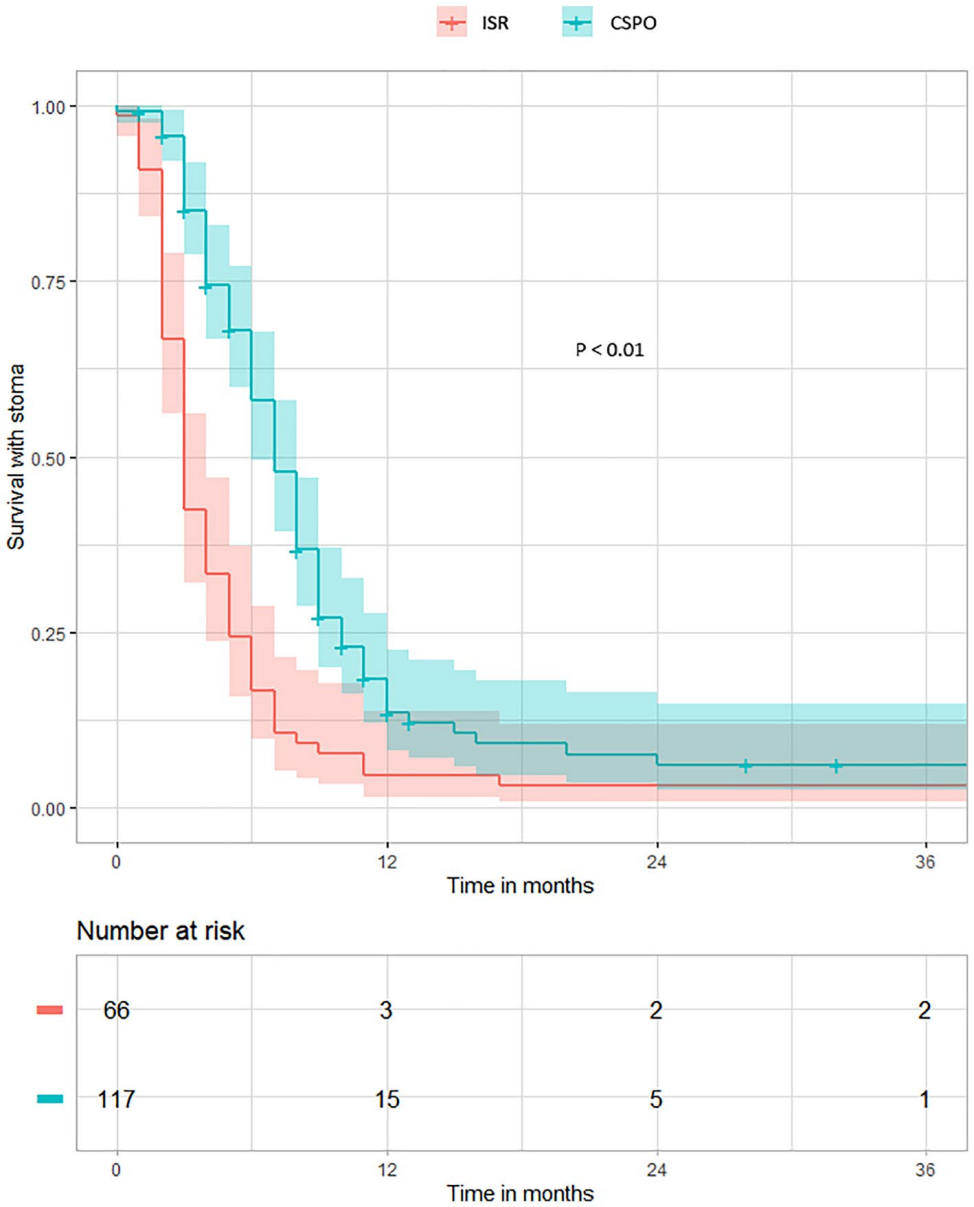
Comparison of the ileostomy reversal between the CSPO and ISR groups with log-rank test

Out of the whole cohort, 97 out of 117 cases in CSPO and 64 out of 66 cases in ISR had the ileostomy reversed (Fig. 2a). Univariable analysis with the age, gender, BMI, type of operation (CSPO vs. ISR), pT stage, pN stage, tumor position, tumor differentiation, preoperative chemoradiotherapy, and postoperative chemotherapy led to the selection of the following variables for multivariable Cox regression analysis: CSPO procedure, age, pT stage, preoperative chemoradiotherapy, tumor differentiation, and postoperative chemotherapy (Supplementary material Table 1). Multivariable Cox regression analysis showed that CSPO contributed to a longer time between primary surgery and stoma reversal (hazard ratio = 0.427, *p* < 0.001, Table 2, Fig. 3). In addition, pT stage, preoperative chemoradiotherapy, and postoperative chemotherapy also significantly contributed to a longer time between primary surgery and stoma reversal (Table 2).

**Table 2:** Multivariable Cox regression analysis of factors affecting stoma reversal free survival

Independent variables	Hazard ratio (HR)^a^	95% CI of HR	*p*
CSPO procedure	0.427	0.293–0.621	< 0.001
Age (years old)	1.022	1.006–1.038	0.007
pT stage	0.590	0.475–0.732	< 0.001
Preoperative chemoradiotherapy	0.671	0.452–0.994	0.047
Tumor differentiation			
High	1.289	0.636–2.613	0.481
Moderate	Reference		
Low	1.120	0.658–1.908	0.677
Postoperative chemotherapy	0.658	0.458–0.946	0.024

^a^HR: exp(coef)

In the CSPO and ISR groups, there were 102 and 66 patients with a follow-up over 12 months after primary surgery, respectively (Fig. 2b). The stoma reversal rates within 1 year after surgery were comparable between CSPO (94/102) and ISR (64/66) (92.16% vs. 96.97%, *p* = 0.318). Univariable logistic regression analysis showed that CSPO did not have a significant difference in ileostomy reversal rate at 12 months (Supplementary material Table 2, *p* = 0.214). In the CSPO group, eight cases could not be reversed within 1 year, including two patients of older age, four patients rejecting reversal, and two patients with radiation-induced pelvic fibrosis who were still receiving conservative treatment at 1 year of follow-up. In the ISR group, two patients failed to be reversed within 1 year, including one patient with postoperative liver metastases and one case rejecting reversal.

### Comparison of anal function

The postoperative follow-up length was not significantly different between CSPO and ISR (40.92 ± 27.10 months vs. 36.74 ± 18.95 months, *p* = 0.226).

Eighty-seven cases from the CSPO group and forty cases from the ISR group finished the questionnaire on anal function (Fig. 2a). Out of those who finished the questionnaire about anal function, the CSPO group had a higher VAS satisfaction with anal function (6.69 ± 2.77 vs. 5.39 ± 2.99, *p* = 0.006), but a comparable LARS and Wexner score in comparison to ISR (Table 3).

**Table 3:** Wexner and LARS score and satisfaction with anal function and quality of life in two groups

Independent variables	CSPO	ISR	*p*
Satisfaction with anal function^a^	6.69 ± 2.77	5.39 ± 2.99	0.006^#^
Wexner incontinence score	7.89 ± 4.57	6.9 ± 4.87	0.284
LARS score	30.57 ± 7.70	29.68 ± 6.83	0.510
Satisfaction with quality of life^a^	7.62 ± 2.13	6.73 ± 2.55	0.058

^#^*p* < 0.05

^a^Range 0–10

After univariable linear regression analysis (Supplementary material Table 3), CSPO procedure, tumor position, preoperative chemoradiotherapy, reversal for longer than 12 months, and postoperative chemotherapy were included in the multivariable linear regression analysis. Multivariable analysis showed that CSPO significantly contributed to a higher anal function satisfaction (beta coefficient = 1.752, *p* < 0.001). Preoperative chemoradiotherapy significantly contributed to a lower anal function satisfaction (beta coefficient = − 1.378, *p* = 0.008) (Table 4).

**Table 4:** Multivariable analysis of factors affecting postoperative anal function

Independent variables	Coefficient	95% CI of coefficient	*p*
Multivariable linear regression analysis of factors affecting VAS satisfaction on anal function			
CSPO procedure	1.752	0.776–2.728	< 0.001
Preoperative chemoradiotherapy	− 1.378	− 2.394 to − 0.361	0.008
Tumor position (cm)	− 0.146	− 0.709 to 0.417	0.609
Reversal longer than 12 months	− 0.777	− 2.252 to 0.697	0.299
Multivariable linear regression analysis of factors affecting LARS score^a^			
CSPO procedure	1.278	− 69.464 to 72.020	0.972
Age	− 1.933	− 4.808 to 0.941	0.186
Gender			
Female	Reference		
Male	65.090	− 1.846 to 175.484	0.057
Preoperative chemoradiotherapy	103.540	31.605–175.484	0.005
Multivariable linear regression analysis of factors affecting Wexner score			
CSPO procedure	0.556	− 1.225 to 2.337	0.538
Preoperative chemoradiotherapy	2.187	0.369–4.006	0.019

Variables were selected on the basis of univariable analysis (Supplementary material) and clinical experience

^a^LARS score^1.8 after transformation for normally distribution of the outcome

Univariable (Supplementary material Tables 4, 5) and multivariable linear regression analysis (Table 4) showed that CSPO did not significantly contribute to different LARS and Wexner scores.

### Comparison of quality of life

Regarding VAS satisfaction with the quality of life, the CSPO group had higher scores (7.62 ± 2.13 vs. 6.73 ± 2.55, *p* = 0.058, Table 3) than the ISR group, but the difference was not significantly different. After univariable linear regression analysis (Supplementary material Table 6), the following variables were included in the multivariable linear regression analysis for factors affecting VAS satisfaction with the quality of life: CSPO procedure, preoperative chemoradiotherapy, tumor position, pT stage, and tumor differentiation. Multivariable analysis showed that CSPO significantly contributed to higher satisfaction with the quality of life (beta coefficient = 1.219, *p* = 0.005). In addition, preoperative chemoradiotherapy and pT stage also significantly contributed to lower satisfaction with quality of life (Table 5).

**Table 5:** Multivariable analysis of factors affecting VAS satisfaction with quality of life and global health

Independent variables	Coefficient	95% CI of coefficient	*p*
Multivariable linear regression analysis of factors affecting VAS satisfaction on quality of life			
CSPO procedure	1.219	0.374–2.064	0.005
Preoperative chemoradiotherapy	− 1.436	− 2.335 to − 0.536	0.002
Tumor position (cm)	− 0.304	− 0.791 to 0.183	0.219
pT stage	− 0.563	− 1.052 to − 0.075	0.024
Tumor differentiation			
High	0.271	− 1.530 to 2.071	0.766
Moderate	Reference		
Low	− 1.130	− 2.325 to 0.066	0.064
Multivariable linear regression analysis of factors affecting global health (QL2)^a^			
CSPO procedure	− 152.700	− 799.196 to 493.804	0.641
Age	− 22.540	− 48.293 to 3.207	0.086
pT stage	− 118.61	− 505.771 to 268.556	0.546
Tumor differentiation			
High	1527.510	302.386–2752.644	0.015
Moderate	Reference		
Low	− 611.040	− 1506.154 to 284.068	0.179

Variables were selected on the basis of univariable analysis (*p* < 0.15) and clinical experience

^a^QL2^1.9 after transformation for normally distribution of the outcome

**Fig. 4:**
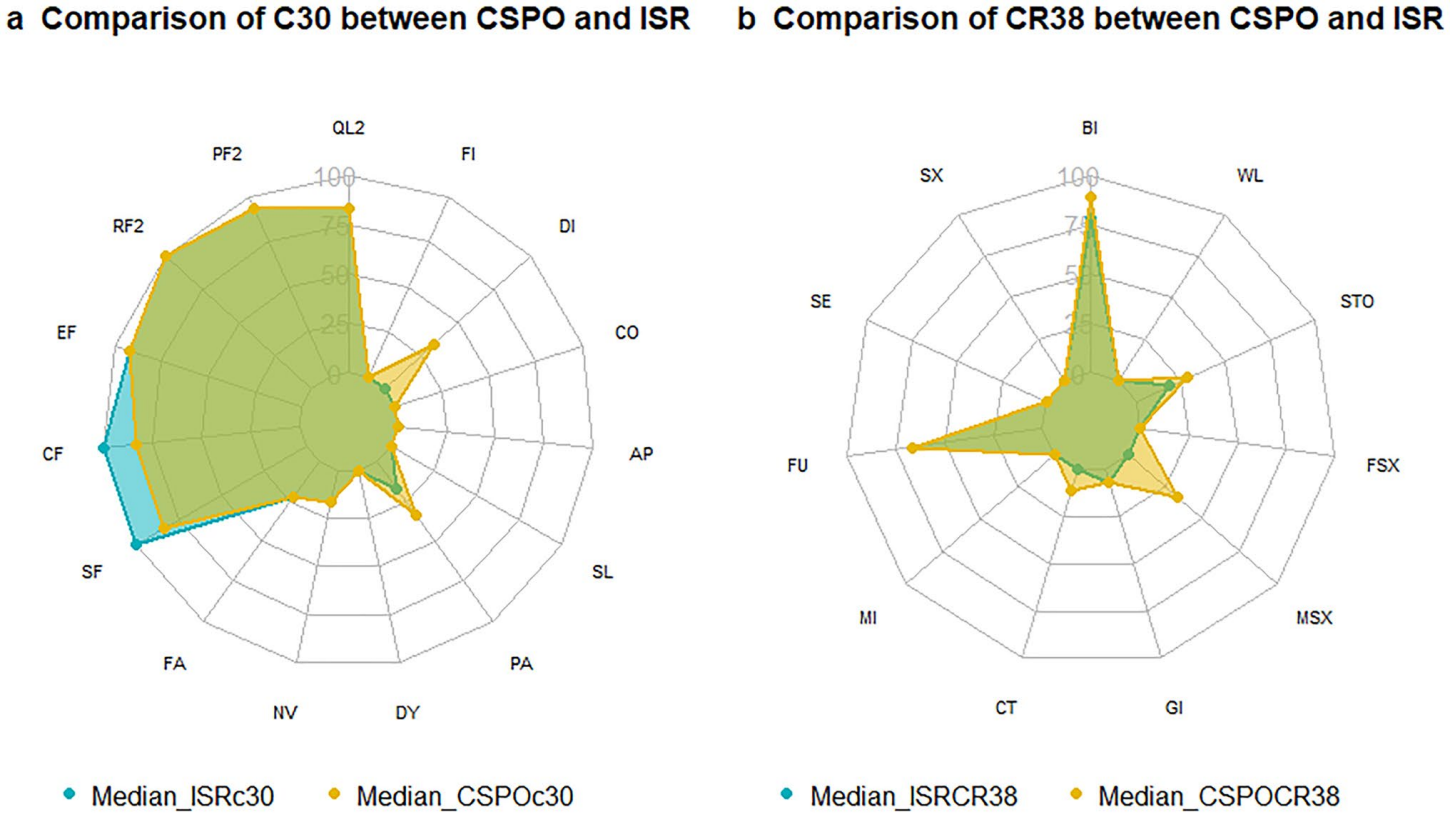
Comparison of quality of life between CSPO (yellow) and ISR (blue) groups regarding **a** EORTC QLQ-C30 and **b** EORTC QLQ-CR38

Regarding the global health (Supplementary material Table 7 for univariable analysis; Table 5 for multivariable analysis) and specific domains of the EORTC QLQ-C30 and QLQ-CR38 (Table 6, Fig. 4), CSPO was not significantly different from ISR.

**Table 6:** Quality of life according to EORTC QLQ-C30 and EORTC QLQ-CR38

Domains	CSPO	ISR
C30		
QL2 (global health)^a^	83.33 (66.67–83.33)	83.33 (66.67–95.83)
PF2 (physical function)^a^	93.33 (86.67–100)	93.33 (86.67–100)
RF2 (role function)^a^	100 (66.67–100)	100 (75–100)
EF (emotional function)^a^	91.67 (75–100)	91.67 (75–100)
CF (cognitive function)^a^	83.33 (83.33–100)	100 (83.33–100)
SF (social function)^a^	83.33 (66.67–100)	100 (66.67–100)
FA (fatigue)^b^	22.22 (0–33.33)	22.22 (0–33.33)
NV (nausea and vomiting)^b^	16.67 (16.67–33.33)	16.67 (16.67–33.33)
DY (dyspnea)^b^	0 (0–0)	0 (0–0)
PA (pain)^b^	33.33 (16.67–50)	16.67 (16.67–33.33)
SL (insomnia)^b^	0 (0–33.33)	0 (0–33.33)
AP (appetite loss)^b^	0 (0–0)	0 (0–33.33)
CO (constipation)^b^	0 (0–33.33)	0 (0–33.33)
DI (diarrhea)^b^	33.33 (0–33.33)	0 (0–33.33)
FI (financial difficulties)^b^	0 (0–33.33)	0 (0–0)
CR38		
BI (body image)^a^	88.89 (66.67–100)	77.78 (66.67–100)
SX (sexual function)^a^	0 (0–33.33)	0 (0–16.67)
SE (sexual enjoyment)^a^	0 (0–33.33)	0 (0–33.33)
FU (future perspective)^a^	66.67 (66.67–100)	66.67 (66.67–100)
MI (micturition problem)^b^	0 (0–22.22)	0 (0–11.11)
CT (chemotherapy side effect)^b^	11.11 (0–22.22)	0 (0–11.11)
GI (symptoms GI)^b^	6.67 (6.67–20)	6.67 (0–13.33)
MSX (male sexual problem)^b^	33.33 (0–50)	0 (0–29.17)
FSX (female sexual problem)^b^	0 (0–33.33)	0 (0–0)
STO (stoma-related problem)^b^	28.57 (9.52–50)	19.05 (11.91–26.19)
WL (weight loss)^b^	0 (0–33.33)	0 (0–33.33)

^a^Functions: range 0–100; 0, lowest function; 100, best function

^b^Symptoms: range 0–100; 0, no symptoms; 100, most severe symptoms

## Discussion

Patients in the CSPO group had lower rectal tumor positions, a higher percentage of male patients, and more preoperative radiotherapy in the current study, which might have led to higher operation difficulty and more blood loss during surgery. Though the CSPO group had comparable Wexner score, LARS score, and quality of life according to EORTC-QLQ-C30 and EORTC-QLQ-CR38 compared to the ISR group, CSPO contributed positively to higher satisfaction regarding anal function and quality of life.

### Surgery-related characteristic comparison

Compared with the ISR group, the CSPO group had a higher proportion of male patients. Male patients have a narrower pelvis compared with female patients, and therefore the operative field was more limited, which in general increases the difficulty of the operation in CSPO [18]. In addition, the CSPO group had a lower tumor position than the ISR group, which also increased the operation difficulty during dissection in the pelvic cavity [18]. The CSPO group also utilized more preoperative chemoradiotherapy in the current study. This might increase fibrosis and swelling in the areas exposed to radiation, which might make the operation more difficult to perform and cause more blood loss in the current study.

Before anastomosis in CSPO, we gradually and slowly dilate the anus with 3–4 fingers to minimize damage to the anus during stapler insertion. The 25-mm staplers were utilized in CSPO, which might be slightly smaller than the stapler used in the Western population, as the size of the Asian population is generally smaller. The 25-mm staplers can preserve more rectal wall, dentate line, and internal anal sphincter during anastomosis, allowing for better preservation of anal function after the operation. Previous literature also demonstrated the safety and effectiveness of 25-mm staplers [6, 19].

CSPO also had a higher laparotomy rate and had one conversion to open surgery, probably as a result of more difficulty during the operation. Besides, the CSPO group also had a shorter duration of operation which might be because of a higher percentage of laparotomy.

Pathological results showed that all patients in both groups achieved R0 resection, and the numbers of lymph nodes retrieved were also similar, which indicated that the surgical pathological resection effects in both groups were similar.

The postoperative hospital stay was significantly shorter in the CSPO group, which might be because the CSPO group had less dissection in the intersphincteric space, which led to less trauma to the nerves, internal anal sphincter, and dentate line and therefore probably less pain. Besides, size 25 mm circular staplers in the CSPO group might also decrease damage to the anal canal tissue when inserting the stapler into the anus [20], which might have led to less postoperative pain in the anal canal.

The CSPO group received a higher rate of postoperative chemotherapy. This is perhaps because we usually administer postoperative chemoradiotherapy according to the pathological results, surgical conditions, and patients’ willingness and general condition. The CSPO group has a lower tumor position and a higher percentage of male patients, which may have led to a higher rate of postoperative chemoradiotherapy than in the ISR group.

### Ileostomy reversal

A British study on 6582 patients with rectal cancer undergoing anterior resection and prophylactic ileostomy showed that 14.6% of patients failed to be reversed 5 years after surgery [21]. However, a Swedish study showed that 21% of 140 patients with rectal cancer who underwent prophylactic ileostomy did not receive stoma reversal 12 years after the primary operation [22]. The ileostomy reversal rate 12 months after surgery in the CSPO group was not significantly different from the ISR group (92.2% vs. 97%) and much higher than that reported in the aforementioned literature [21, 22]. However, the CSPO group had a longer interval between primary surgery and stoma reversal. More preoperative chemoradiotherapy in the CSPO group might have led to a longer time between primary surgery and ileostomy compared to the ISR group [23].

### Anal function and quality of life

The follow-up time in CSPO and ISR was comparable (40.92 ± 27.10 vs. 36.74 ± 18.95 months, *p* = 0.226). According to the literature, anal function was usually stable 1 year after ileostoma reversal, so our follow-up length is sufficient to measure anal function. The average VAS satisfaction with anal function in the CSPO group was 6.69, which was significantly higher than 5.39 for ISR in the current study, and also higher than the 5 points reported by Zhang et al. for ISR [11]. In multivariable analysis, after adjustment for other confounding factors, CSPO can still improve satisfaction with anal function. This might be because in CSPO more dentate line, rectal wall, and internal anal sphincter can be preserved and the nerves in the intersphincteric space can be protected. In multivariable analysis, preoperative chemoradiotherapy can decrease the VAS anal function satisfaction and increase the LARS/Wexner score, which is consistent with Ito et al.’s finding that preoperative chemoradiotherapy can negatively influence the postoperative anal function [24]. Preoperative chemoradiotherapy will reduce patients’ satisfaction with anal function, and the CSPO group had a higher proportion of preoperative chemoradiotherapy, but had higher satisfaction with anal function after surgery, thus highlighting the advantages of CSPO in anal function protection.

Regarding the quality of life, after adjusting for confounders, we found that CSPO contributed to significantly higher satisfaction with the quality of life. This might be due to the higher satisfaction with anal function in the CSPO group as we have discussed before.

In none of the Wexner score, LARS score, different domains of the EORTC-QLQ-C30, or EORTC-QLQ-CR38 questionnaires did CSPO significantly differ from ISR in the multivariable analysis. However, the CSPO group still achieved higher satisfaction with postoperative anal function and quality of life. This might be because despite the higher difficulty of sphincter preservation and the negative influence on anal function and quality of life from the preoperative chemoradiotherapy in the CSPO group, the quantitative results such as the Wexner score, LARS score, and scores of EORTC questionnaire are still comparable between CSPO and ISR. These comparable results will improve the subjective psychological satisfaction of the patients in the CSPO group.

### Clinical implication

This study has several implications for clinical practice. By comparing CSPO and ISR, we distinguished the pros and cons of these two surgical procedures. On one hand, in the current cohort, CSPO preserves the anus when the tumor position was lower than in the ISR group. Meanwhile, CSPO can improve satisfaction with anal function and quality of life. This is the strength of CSPO in comparison to ISR. Considering the fact that CSPO and ISR share similar oncological and perioperative safety according to our previous research [7], the functional outcome seems to be quite important when choosing between the two procedures. On the other hand, to achieve more sphincter preservation, CSPO utilized more preoperative chemoradiotherapy, which can help in downstaging the tumor, decreasing the tumor size, and increasing the tumor position, especially in male patients with a narrow pelvic cavity. However, preoperative chemoradiotherapy might also bring some side effects, like postponed ileostomy reversal as was found in the current study, even though the reversal rate was not significantly different between both groups 12 months after surgery. Before choosing between CSPO and ISR, a comprehensive evaluation should be carried out regarding the patients’ expectations about anal function and quality of life in addition to the physical strength for tolerating radiotherapy. For some younger patients who are more resistant to chemoradiotherapy and have higher requirements about anal function and quality of life, CSPO may be an alternative choice for patients with very low rectal cancer.

This study has some limitations. First, this is a retrospective study and some data are missing. Second, the number of patients is relatively small. CSPO and ISR have strict indications. Once we have made a recommendation of operation for the patient, the patient decides whether to follow our recommendation according to their own circumstances. The limited volume of patients in the study may be due to patient choice. However, we compared the functional outcome and quality of life between the two surgical procedures from various perspectives. These include ileostomy reversal rate, Wexner incontinence score, VAS satisfaction with anal function, VAS satisfaction with quality of life, and different domains of quality of life in the EORTC QLQ-C30 and EORTC QLQ-CR38 questionnaires. Despite the data missing as a result of the retrospective study design, our study provides valuable insights into the functional outcomes and quality of life for patients who underwent CSPO compared to ISR. By analyzing the results and comparing them with recent literature, we can better understand the benefits and limitations of each procedure and contribute to the ongoing development of surgical techniques for treating very low rectal cancer. For the aforementioned results, we employed multivariable analysis to adjust for potential confounders. This analysis revealed that CSPO has higher VAS satisfaction with anal function and quality of life. However, concerning the reversal rate, Wexner score, LARS score, and EORTC CR-38 and EORTC-C30, multivariate analysis did not show a significant difference between the two procedures. As a result, we concluded that CSPO is non-inferior to ISR in terms of anal function and quality of life issues, rather than being superior to ISR. Further studies with larger sample sizes are still necessary to confirm our findings and establish stronger evidence for the benefits of CSPO in comparison to ISR. Third, the tumor position, preoperative treatment, and genders were different between the CSPO and ISR groups. We did not use propensity score matching to balance the distribution of these three parameters, because these three parameters were considered to be the characteristics of the surgical procedure, as described previously. Alternatively, we used multivariable regression analysis to adjust for the possible confounding factors for the surgical procedures in the current study. Fourth, the satisfaction with the quality of life and anal function is still subjective, thus we utilized the same questionnaire online in two centers to decrease the influence from communication.

## Conclusion

The CSPO group has lower rectal tumor positions, a higher percentage of male patients, and more preoperative (chemo)radiotherapy in the current study population, which might lead to a more difficult operation and therefore more blood loss during the operation. Despite this fact, CSPO had shorter postoperative stays and contributed to higher satisfaction with anal function and quality of life than ISR. CSPO may be an alternative choice for patients with very low rectal cancer in better physical health and who have higher requirements for anal function and quality of life.

### Supplementary Information

Below is the link to the electronic supplementary material.

Supplementary file1 (DOCX 47 kb)

## Data Availability

Data will be available on reasonable request to the authors.
