# Supplementary material for: Comparison of anal function and quality of life after conformal sphincter preservation operation and intersphincteric resection of very low rectal cancer: a multicenter, retrospective, case–control analysis
Source: Tech Coloproctol. 2023 May 29;27(12):1275–87. doi: 10.1007/s10151-023-02819-w (PMC10638180; doi:10.1007/s10151-023-02819-w)
Supplement: Supplementary file 1 — Supplementary file1 (DOCX 47 kb) [file 10151_2023_2819_MOESM1_ESM.docx]

| **Supplementary material table 1. Univariable Cox regression analyses of factors that could influence ileostomy reversal in two groups (n = 183 )** | | | | |
| --- | --- | --- | --- | --- |
| **Independent variables** | **Hazard ratio (HR)** | **95% CI HR** | | ***p*** |
|  |  | **Lower bound** | **Upper bound** |  |
| **Type of operation (CSPO)** | 0.402 | 0.292 | 0.555 | **< 0.001** |
| **Age** | 1.015 | 0.999 | 1.03 | **0.052** |
| Gender |  |  |  |  |
| Female | Reference |  |  |  |
| Male | 0.939 | 0.681 | 1.293 | 0.697 |
| BMI | 1.026 | 0.973 | 1.081 | 0.343 |
| **Preoperative chemoradiotherapy** | 0.671 | 0.461 | 0.977 | **0.037** |
| Tumor position (cm) | 1.049 | 0.922 | 1.193 | 0.467 |
| Tumor diameter (cm) | 0.997 | 0.879 | 1.131 | 0.962 |
| **pT stage** | 0.701 | 0.578 | 0.852 | **< 0.001** |
| pN stage |  |  |  |  |
| N0 | Reference |  |  |  |
| N1 | 0.786 | 0.507 | 1.219 | 0.282 |
| N2 | 1.032 | 0.557 | 1.913 | 0.920 |
| Tumor differentiation |  |  |  |  |
| High | 1.895 | 0.991 | 3.626 | 0.053 |
| Moderate | Reference |  |  |  |
| Low | 0.949 | 0.564 | 1.596 | 0.843 |
| **Postoperative chemotherapy** | 0.441 | 0.321 | 0.606 | **< 0.001** |

| **Supplementary material table 2. Univariable logistic regression analyses of factors that could influence ileostomy reversal in patients with follow up for over 12 months (n = 168 )** | | | | |
| --- | --- | --- | --- | --- |
| **Independent variables** | **Odds ratio (OR)** | **95% CI OR** | | ***p*** |
|  |  | **Lower bound** | **Upper bound** |  |
| CSPO procedure | 0.367 | 0.076 | 1.786 | 0.214 |
| Age | 1.037 | 0.979 | 1.098 | 0.212 |
| Gender |  |  |  |  |
| Female | Reference |  |  |  |
| Male | 1.59 | 0.442 | 5.721 | 0.478 |
| BMI | 0.997 | 0.809 | 1.228 | 0.976 |
| Preoperative chemoradiotherapy | 0.411 | 0.110 | 1.541 | 0.187 |
| Tumor position (cm) | 0.693 | 0.434 | 1.108 | 0.126 |
| Tumor diameter (cm) | 1.260 | 0.742 | 2.140 | 0.393 |
| pT stage | 0.909 | 0.395 | 2.094 | 0.823 |
| pN stage |  |  |  |  |
| N0 | Reference |  |  |  |
| N1 | 1.756 | 0.213 | 14.510 | 0.601 |
| N2 | 8.462e+06 | 0 | Inf | 0.994 |
| Tumor differentiation |  |  |  |  |
| High | 7.885e+06 | 0 | Inf | 0.994 |
| Moderate | Reference |  |  |  |
| Low | 1.091 | 0.130 | 9.181 | 0.936 |
| Postoperative chemotherapy |  |  |  |  |
| No | Reference |  |  |  |
| Yes | 0.478 | 0.130 | 1.762 | 0.268 |

| **Supplementary material table 3. Univariable linear regression analyses of factors that influencing VAS satisfaction on anal function (n = 127)** | | | | |
| --- | --- | --- | --- | --- |
| **Independent variables** | **Beta coefficient**^†^ | **95% CI beta** | | ***p*** |
|  |  | **Lower bound** | **Upper bound** |  |
| **CSPO procedure** | 1.57 | 0.497 | 2.646 | **0.004** |
| Age | -0.019 | -0.065 | 0.026 | 0.405 |
| Gender |  |  |  |  |
| Female | Reference |  |  |  |
| Male | -0.115 | -1.20 | 0.97 | 0.834 |
| BMI | -0.027 | -0.194 | 0.141 | 0.753 |
| **Preoperative chemoradiotherapy** | -1.109 | -2.26 | 0.039 | 0.058 |
| **Tumor position (cm)** | -0.504 | -1.06 | 0.05 | 0.070 |
| Tumor diameter (cm) | 0.039 | -0.354 | 0.433 | 0.843 |
| pT stage | -0.20 | -0.85 | 0.44 | 0.537 |
| pN stage |  |  |  |  |
| N0 | Reference |  |  |  |
| N1 | 0.678 | -0.716 | 2.072 | 0.337 |
| N2 | -0.136 | -2.799 | 2.526 | 0.919 |
| Tumor differentiation |  |  |  |  |
| High | 1.767 | -0.471 | 4.005 | 0.121 |
| Moderate | Reference |  |  |  |
| Low | -1.005 | -2.588 | 0.577 | 0.211 |
| Postoperative chemotherapy | 0.778 | -0.135 | 1.690 | 0.097 |
| Reversal > 12 months | -1.284 | -2.976 | 0.408 | 0.136 |
| ^†^Unstandardised beta coefficient | | | | |

| **Supplementary material table 4. Univariable linear regression analyses of factors influencing the LARS score* (n = 127)** | | | | |
| --- | --- | --- | --- | --- |
| **Independent variables** | **Beta coefficient**^†^ | **95% CI beta** | | ***p*** |
|  |  | **Lower bound** | **Upper bound** |  |
| CSPO procedure | 29.590 | -41.753 | 100.925 | 0.413 |
| **Age** | -2.612 | -5.532 | 0.309 | 0.079 |
| **Gender** |  |  |  |  |
| Female | Reference |  |  |  |
| Male | 67.900 | -0.883 | 136.685 | 0.053 |
| BMI | 3.294 | -7.474 | 14.061 | 0.546 |
| **Preoperative chemoradiotherapy** | 105.580 | 32.905 | 178.260 | **0.005** |
| Tumor position (cm) | -2.252 | -38.341 | 33.836 | 0.902 |
| Tumor diameter (cm) | -10.430 | -35.744 | 14.883 | 0.416 |
| pT stage | -25.890 | -67.408 | 15.626 | 0.219 |
| pN stage |  |  |  |  |
| N0 | Reference |  |  |  |
| N1 | 16.640 | -73.327 | 106.606 | 0.715 |
| N2 | -53.940 | -225.798 | 117.927 | 0.536 |
| Tumor differentiation |  |  |  |  |
| High | 53.460 | -92.617 | 199.540 | 0.470 |
| Moderate | Reference |  |  |  |
| Low | -38.460 | -141.749 | 64.836 | 0.463 |
| Postoperative chemotherapy | 13.010 | -53.656 | 79.671 | 0.700 |
| Reversal > 12 months | -79.010 | -187.951 | 29.921 | 0.154 |
| ^†^Unstandardised beta coefficient | | | | |
| *LARS score^1.8 after transformation for normally distribution of the outcome. | | | | |

| **Supplementary material table 5. Univariable linear regression analyses of factors influencing the Wexner score (n = 127)** | | | | |
| --- | --- | --- | --- | --- |
| **Independent variables** | **Beta coefficient**^†^ | **95% CI beta** | | ***p*** |
|  |  | **Lower bound** | **Upper bound** |  |
| CSPO procedure | 0.985 | -0.779 | 2.749 | 0.271 |
| Age | 0.025 | -0.048 | 0.098 | 0.505 |
| Gender |  |  |  |  |
| Female | Reference |  |  |  |
| Male | -0.094 | -1.824 | 1.636 | 0.914 |
| BMI | 0.112 | -0.154 | 0.379 | 0.406 |
| **Preoperative chemoradiotherapy** | 2.187 | 0.369 | 4.006 | **0.020** |
| Tumor position (cm) | -0.067 | -0.961 | 0.827 | 0.882 |
| Tumor diameter (cm) | 0.398 | -0.227 | 1.022 | 0.210 |
| pT stage | 0.400 | -0.632 | 1.432 | 0.444 |
| pN stage |  |  |  |  |
| N0 | Reference |  |  |  |
| N1 | 0.981 | -1.236 | 3.199 | 0.383 |
| N2 | -2.095 | -6.331 | 2.141 | 0.330 |
| Tumor differentiation |  |  |  |  |
| High | -1.695 | -5.319 | 1.929 | 0.356 |
| Moderate | Reference |  |  |  |
| Low | -0.229 | -2.791 | 2.333 | 0.860 |
| Postoperative chemotherapy | 0.751 | -0.896 | 2.398 | 0.369 |
| Reversal > 12 months | -1.291 | -4 | 1.417 | 0.347 |
| ^†^Unstandardised beta coefficient | | | | |

| **Supplementary material table 6. Univariable linear regression analyses of factors influencing the VAS satisfaction on quality of life (n = 183)** | | | | |
| --- | --- | --- | --- | --- |
| **Independent variables** | **Beta coefficient**^†^ | **95% CI beta** | | ***p*** |
|  |  | **Lower bound** | **Upper bound** |  |
| **CSPO procedure** | 0.896 | 0.037 | 1.754 | **0.041** |
| Age | -0.012 | -0.049 | 0.023 | 0.489 |
| Gender |  |  |  |  |
| Female | Reference |  |  |  |
| Male | -0.143 | -0.994 | 0.709 | 0.741 |
| BMI | -0.081 | -0.212 | 0.049 | 0.220 |
| **Preoperative chemoradiotherapy** | -0.985 | -1.884 | -0.085 | **0.032** |
| **Tumor position (cm)** | -0.387 | -0.822 | 0.048 | 0.081 |
| Tumor diameter (cm) | 0.088 | -0.221 | 0.397 | 0.574 |
| **pT stage** | -0.410 | -0.915 | 0.095 | 0.110 |
| pN stage |  |  |  |  |
| N0 | Reference |  |  |  |
| N1 | 0.167 | -0.927 | 1.261 | 0.763 |
| N2 | -1.156 | -3.247 | 0.934 | 0.276 |
| **Tumor differentiation** |  |  |  |  |
| High | 1.171 | -0.584 | 2.927 | 0.189 |
| Moderate | Reference |  |  |  |
| Low | -1.067 | -2.308 | 0.175 | 0.092 |
| Postoperative chemotherapy | 0.392 | -0.419 | 1.203 | 0.340 |
| Reversal > 12 months | -0.134 | -1.478 | 1.209 | 0.844 |
| ^†^Unstandardised beta coefficient | | | | |

| **Supplementary material table 7. Univariable linear regression analyses of factors influencing global health (QL2) from C30* (n = 183)** | | | | |
| --- | --- | --- | --- | --- |
| **Independent variables** | **Beta coefficient**^†^ | **95% CI beta** | | ***p*** |
|  |  | **Lower bound** | **Upper bound** |  |
| **CSPO procedure** | -37.190 | -691.428 | 617.054 | 0.911 |
| **Age** | -21.220 | -47.548 | 5.113 | 0.113 |
| Gender |  |  |  |  |
| Female | Reference |  |  |  |
| Male | -141.400 | -751.305 | 468.408 | 0.647 |
| BMI | 8.882 | -89.135 | 106.900 | 0.858 |
| Preoperative radiotherapy | -456.400 | -1106.112 | 193.241 | 0.167 |
| Tumor position (cm) | 89.840 | -218.200 | 397.872 | 0.565 |
| Tumor diameter (cm) | 32.670 | -193.386 | 258.720 | 0.775 |
| **pT stage** | -298.600 | -666.526 | 69.373 | 0.111 |
| pN stage |  |  |  |  |
| N0 | Reference |  |  |  |
| N1 | 372 | -434.677 | 1178.600 | 0.363 |
| N2 | -516.300 | -2096.475 | 1063.896 | 0.519 |
| **Tumor differentiation** |  |  |  |  |
| High | 1515.200 | 281.159 | 2749.152 | **0.017** |
| Moderate | Reference |  |  |  |
| Low | -575.500 | -1476.220 | 325.223 | 0.209 |
| Postoperative chemotherapy | -367.1 | -952.958 | 218.837 | 0.218 |
| Reversal > 12 months | 289.500 | -511.397 | 1090.385 | 0.476 |
| ^†^Unstandardised beta coefficient | | | | |
| *QL2^1.9 after transformation for normally distribution of the outcome. | | | | |
